# Doomed before Birth

**Published:** 1906-04-28

**Authors:** 


### DOOMED BEFORE BIRTH.

Miss Frances Zanetti, the inspector appointed under the Infant Life Protection Act by the Chorlton Board of Guardians, has in her last report put forward some strong reasons for extending the provisions of the Act to all nurse children. At present anyone can take a single child to nurse without being liable to inspection, and it is just in those cases that children pine and die in such a fashion that the law cannot say that they are done to death, although it is known that care and proper feeding might have saved them. Where a single child is taken, the foster mother is often a friend of the mother, but Miss Zanetti is convinced that the friend of the mother of an illegitimate child—and most nurse children are illegitimate—is not necessarily the best nurse. Her sympathies are with the mother, to whom the child is a burden and a disgrace, and she is not too keen about keeping it alive. Moreover, these children are usually handicapped before birth. The medical officers of workhouses say that they have an excessive proportion of difficult obstetric cases because the mothers have so often used one means or another to procure abortion. Even when they have been unsuccessful the constitution of the children is injured, and they would require extra care in their early years if they are to be reared at all. If such a chiia is put in the care of an ignorant woman, even if she is kind and well-meaning, its chances are poor. The child may share the food of the family—and it might be said that one cannot ask for more—but that food, though fit enough for healthy children, is not fitted for a baby whose ancestral health history is often bad and who may have been nearly poisoned before its birth. Yet, if only one child is taken no one has the right to interfere unless wilful cruelty is proved. Miss Zanetti's experience is that the better class of women welcome the visits of the inspector, whether they take in one child or more, and the testimony of foster mothers is that no person would object to inspection unless she had something to hide. Miss Zanetti says: "I have known several instances this year in which women who have been under inspection, and who have known that their licenses were in danger, have given up both their nurse-children thereby removing their case from the register, and have afterwards taken one child and uefied interference." It is to be hoped that before long the law will be so amended as to prevent this evasion of its intentions.

